# 2-Hydroxyglutarate in Acute Myeloid Leukemia: A Journey from Pathogenesis to Therapies

**DOI:** 10.3390/biomedicines10061359

**Published:** 2022-06-09

**Authors:** Vittoria Raimondi, Giulia Ciotti, Michele Gottardi, Francesco Ciccarese

**Affiliations:** 1 Department of Surgery, Oncology and Gastroenterology, University of Padua, 35128 Padova, Italy; 2 Onco Hematology, Department of Oncology, Veneto Institute of Oncology IOV–IRCCS, 31033 Castelfranco Veneto, Italy; giulia.ciotti@iov.veneto.it (G.C.); michele.gottardi@iov.veneto.it (M.G.); 3 Immunology and Molecular Oncology Unit, Veneto Institute of Oncology IOV–IRCCS, 35128 Padova, Italy; francesco.ciccarese@iov.veneto.it

**Keywords:** AML, 2-HG, diagnosis, therapy

## Abstract

The oncometabolite 2-hydroxyglutarate (2-HG) plays a key role in differentiation blockade and metabolic reprogramming of cancer cells. Approximatively 20–30% of acute myeloid leukemia (AML) cases carry mutations in the isocitrate dehydrogenase (IDH) enzymes, leading to a reduction in the Krebs cycle intermediate α-ketoglutarate (α-KG) to 2-HG. Relapse and chemoresistance of AML blasts following initial good response to standard therapy account for the very poor outcome of this pathology, which represents a great challenge for hematologists. The decrease of 2-HG levels through pharmacological inhibition of mutated IDH enzymes induces the differentiation of AML blasts and sensitizes leukemic cells to several anticancer drugs. In this review, we provide an overview of the main genetic mutations in AML, with a focus on IDH mutants and the role of 2-HG in AML pathogenesis. Moreover, we discuss the impact of high levels of 2-HG on the response of AML cells to antileukemic therapies and recent evidence for highly efficient combinations of mutant IDH inhibitors with other drugs for the management of relapsed/refractory (R/R) AML.

## 1. Introduction

Acute myeloid leukemia (AML) is an extremely heterogeneous hematologic malignancy, characterized by clonal expansion of myeloid blasts in the peripheral blood, bone marrow, and/or other tissues [1]. This malignancy comprises most acute leukemias cases affecting the myeloid lineage and accounts for about 20% of all acute leukemias in children and 80% in adults [2,3,4]. In Europe, the incidence of AML is approximately 3.7 per 100,000 persons/year [5], among which 2–3% of cases are pediatric [6,7]. AML is the most lethal leukemia, with 11,180 estimated deaths annually worldwide [8]; its poor prognosis appears correlated to the advanced age of patients, comorbidities, as well as to high-risk genetic features or antecedent hematologic disorders (e.g., myelodysplastic syndrome) [9]. Despite considerable progress having been made in understanding the biological landscape, prognosis remains poor, especially in elderly subjects [10]. In particular, the 5-year overall survival (OS) is about 30%, which decreases along with the increase in age, resulting in less than 10% in over 75 patients (available from https://seer.cancer.gov/explorer/ (accessed on 28 April 2022)).

**Figure 1:**
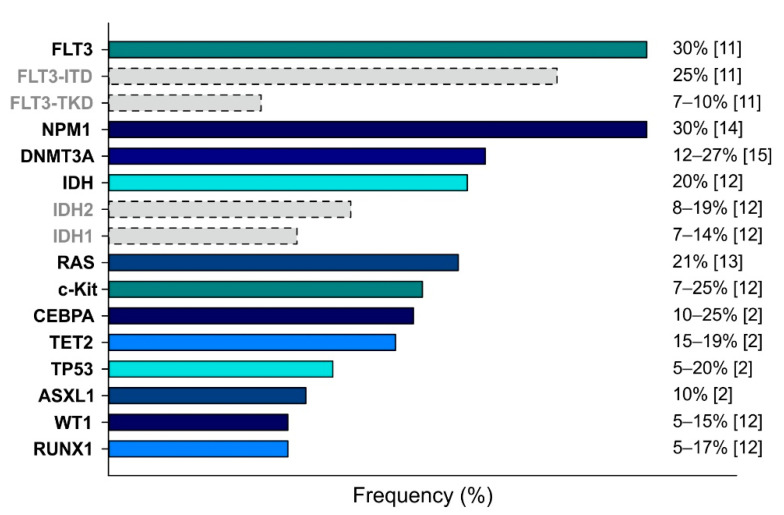
Main genetic mutations detected in AML cases. Mutations are ranked by frequency (indicated on the right). Dotted bars indicate the partial contributes of the specific subtypes.

AML is a highly complex disease characterized by a plethora of cytogenetic alterations and genetic mutations, which are used to classify AML. Figure 1 shows the frequencies of the most relevant mutations described for AML, which affect the following genes: Fms-Like Tyrosine Kinase 3 (FLT3) [11], KIT [12], RAS [13], Nucleophosmin 1 (NPM1) [14], CCAAT Enhancer Binding Protein α (CEBPA) [2], Runt-related transcription factor (RUNX1) [12], DNA methyltransferase 3A (DNMT3A) [15], Isocitrate dehydrogenase (IDH) [12], Ten–eleven translocation 2 (TET2) [2], Additional sex comb-like 1 (ASXL1) [2], Wilms’ tumor 1 (WT1) [12], and Tumor protein 53 (TP53) [2].

The therapeutic approaches are also established on the classification of patients in fit, unfit, and frail. This stratification is based on several features, including age, performance status (which allows identifying higher-risk AML patients independently of age), and comorbidities [9]. Patients less than 70 years old who are low-risk and without comorbidities are usually considered fit and are subjected to standard intensive treatment, which consists of a two-phase regimen that includes an induction and consolidation phase, often followed by allogenic hematopoietic stem cell transplantation (allo-HSCT) [16]. Induction therapy is aimed at reaching a complete remission (CR) through a combination of cytarabine (arabinosylcytosine, ara-C) and an anthracycline (daunorubicin or idarubicin), administered through a “7 + 3” protocol [17]. It can also be implemented with purine analogs (cladribine or fludarabine), which improve patient outcomes by promoting the accumulation of cytarabine triphosphate (ara-CTP) in leukemia cells [18]. However, due to its severe toxicities and side effects, intensive therapy is not recommended for unfit and frail patients [17]. While frail patients seem to benefit more from palliative care alone [17], an important issue in the management of AML is represented by unfit patients, who are not eligible to receive the induction therapy and are subjected to less intensive regimens [19] based on hypomethylating agents (HMA) or low-dose cytarabine (LDAC) [20]. However, the prognosis of these patients is extremely poor as they will not receive consolidation therapy, which is crucial to obtaining a durable disease remission.

Moreover, despite a good response to first-line treatment (more than 70% of cases), most AML patients relapse within 6–9 months after the consolidation phase. Notably, the treatment options for relapsed/refractory (R/R) patients are limited and new strategies are urgently needed. In this scenario, the development of targeted therapies has led to remarkable successes in some genetic subtypes of AML, leading to improved survival and quality of life [21], making the correct identification of genetic alterations and the stratification of AML subtypes of primary importance. Several compounds were developed to treat AML patients who present peculiar genetic mutations.

In this review, we focused on the mutations of IDH1 and IDH2, coding for the cytosolic and the mitochondrial NADP-dependent isocitrate dehydrogenases, respectively [12]. Wild-type IDH enzymes produce NADPH by converting isocitrate to oxalosuccinate and then to α-ketoglutarate (α-KG), which is an essential factor in the tricarboxylic acid (TCA) cycle [22], while mutant IDHs lead to NADPH consumption by converting isocitrate to the oncometabolite 2-hydroxyglutarate (2-HG). A significant accumulation of 2-HG into cells was described in AML patients with IDH mutations [23], causing the inhibition of α-KG-dependent enzymes [24] and the consequent deregulation of the methylation state of DNA and gene expression, which can activate oncogenes and inactivate tumor-suppressor genes. Not only AML, but several different tumor types–such as astrocytoma and glioblastoma–carry mutations of IDH1/2, underlying the critical role of these enzymes in tumorigenesis [25]. Moreover, the levels of 2-HG are reported to be directly proportional to the worst prognosis [26].

In this context, patients with IDH mutations could benefit from pharmacological inhibition of IDH enzymes through specific small molecules. In parallel, pharmacological approaches that target altered molecular pathways were also developed. Indeed, both fit and unfit patients with IDH mutations appear more sensitive to the treatment with venetoclax + azacitidine than the IDH1/2 wild type, achieving higher and more durable response rates and more prolonged median OS [27].

Venetoclax is a BCL-2 inhibitor, recently approved by the Food and Drug Administration (FDA) in combination with HMA to treat de novo AML patients unfit for intensive chemotherapy, where BCL-2 anti-apoptotic protein is usually upregulated and associated with poor prognosis and resistance to chemotherapy [28]. Clinical reports highlight a significant benefit in patients treated with venetoclax + azacitidine compared to azacitidine alone, with an increase in the OS from 9.6 to 14.7 months and a CR of 37.7% vs. 17.9% (*p* < 0.001) [29]. A phase II trial of venetoclax monotherapy was also performed in patients with R/R AML [30].

However, the response to venetoclax-based treatments appears limited in time [31] and the onset of resistant clones suggests that BCL-2 inhibition alone, though effective, was not sufficient for patients with R/R AML. Additionally, in this case, the impact of genetic alterations appears crucial. It is reported that the inactivation of the *TP53* and *BAX* genes are key elements of resistance to venetoclax, explaining venetoclax insensitivity in samples from AML patients who have low expression of these two genes [32].

These observations suggest that it is of primary importance to study the effect of genetic mutations in AML development and pharmacological response, as well as to understand the molecular mechanism of drug resistance to prevent and/or counteract it.

## 2. The Role of 2-HG in AML Pathogenesis

### 2.1. Biochemical Alterations in IDH Mutant Cells

As described above, the accumulation of the oncometabolite 2-HG in AML cells is mainly associated with the mutations of IDH1/2 and their acquired neomorphic activity. Indeed, these mutations, which affect the arginine residues in the catalytic domain (IDH1^R132^, IDH2^R140^, or IDH2^R172^), do not lead to an inactive form of IDH1/2 but allow them to catalyze a new reaction [33,34]. Wild-type IDH1/2 work as homodimers; they bind isocitrate and catalyze its oxidative decarboxylation by using NADP^+^ as a cofactor, with the consequent implementation of the NADPH cellular pool [35]. Conversely, the neomorphic activity derives from the greater affinity of the mutated enzymes for α-KG and NADPH than for isocitrate, which impedes oxidative decarboxylation of isocitrate to α-KG, resulting in the reduction of α-KG to 2-HG [33,36] and consumption of NADPH. In tumors, mutated IDHs heterodimerize with the wild-type forms, overwhelming their activity and inducing changes in the orientation of the catalytic site, which allow the binding of NADPH and α-KG instead of NADP^+^ and isocitrate [33].

2-HG is responsible for the inhibition of enzymes normally activated by α-KG by competitively binding them. In particular, the inhibition of dioxygenases that regulate the cellular epigenetic state can lead to chromatin abnormalities [37,38], thus blocking the cellular differentiation and increasing the proliferation of hematopoietic cells [39]. Moreover, like other enzymes in the tricarboxylic acid cycle (e.g., succinate dehydrogenase, fumarate hydratase), IDHs are involved in synthesizing nucleotides, lipids, and amino acids, suggesting that their alterations could have an important role in tumorigenesis.

### 2.2. Effects of IDH Mutations in Cancer

Mutated IDHs are frequently found in many tumor types, including AML, gliomas, and sarcomas, but the accumulation of 2-HG is also associated with other non-malignant diseases, such as encephalopathy and cardiomyopathy [40]. In particular, about 20% of adult AML patients present early mutations of IDHs [37,41], which are associated with an about 100-fold increase in the levels of 2-HG compared to wild-type IDHs [33,34,42,43], suggesting 2-HG as playing a role as a marker of tumor-associated IDH mutations [42].

The wild-type IDH1/2, together with other enzymes (such as malic enzyme 1, glucose-6-phosphate dehydrogenase and 6-phosphogluconate dehydrogenase), maintain the NADPH pool and redox homeostasis in non-malignant cells [44]. On the other hand, mutations of IDH1/2 shift redox homeostasis in cytosol and mitochondria, respectively, toward the more oxidated state due to the concomitant reduction of α-KG and the presence of high levels of 2-HG (2–600 µmol/L in AML patients’ serum) [45]. Both events are needed to drive the decrease in the NADPH/NADP^+^ ratio, which in turn lead to an increase in reactive oxygen species (ROS) by depleting the main source of electrons used to regenerate the antioxidant systems [34,46]. Moreover, high levels of 2-HG lead to slowed down OXPHOS metabolism and mitochondrial respiration, with consequent leakage of electrons from the respiratory chain and excessive accumulation of mitochondrial superoxide [47].

**Figure 2:**
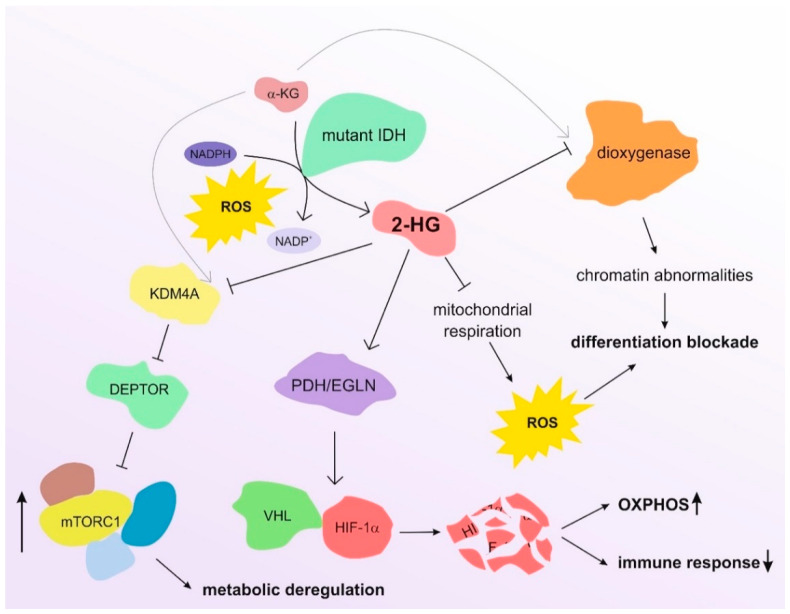
Main molecular effects of 2-HG in AML cells. The reduction of α-KG to 2-HG by mutated IDH enzymes leads to profound alterations in the biology of AML cells. The inhibition of α-KG-dependent dioxygenases–such as TET2 [33]—alters the chromatin architecture, thus rewiring gene expression and inducing differentiation blockade. Moreover, 2-HG leads to metabolic deregulation through the activation of the mTOR pathway and the inhibition of the HIF pathway. The increase in ROS levels resulting from NADPH consumption and altered mitochondrial respiration contributes to the blockade of differentiation and the aggressiveness of AML blasts.

Elevated levels of 2-HG are also associated with the reprogrammed metabolism of many tumor types, such as glioma, glioblastoma, and AML [33,48], and their role in the induction of oxidative stress also affects the crosstalk between tumors and immune cells by inducing the differentiation of naïve T lymphocytes and regulating immune cells [49,50]. In parallel, 2-HG is reported to affect different transcription factors, such as hypoxia-inducible factor (HIF) and mammalian target of rapamycin (mTOR), which are commonly deregulated in cancer [51]. 2-HG can activate the HIF-1α-specific prolyl hydroxylases (PDH/EGLN), which in turn promotes recognition of HIF-1α by von Hippel–Lindau (VHL) E3 ligase and its consequent proteasomal degradation [39,52,53]. This impairment of the HIF signaling contributes to the shift from glycolysis to OXPHOS in malignant cells and reduces the differentiation of T helper 17 cells [54], counteracting the immune response against tumor cells. On the other hand, 2-HG promotes the mTOR pathway by inhibiting KDM4A, a member of the Jumonji protein family, which demethylates lysine residues using α-KG as co-substrate [55]. KDM4A inhibition leads to reduced levels and stability of DEPTOR by allowing the activity of β-TrCP ubiquitin ligases. DEPTOR is a negative regulator of mTORC1/2, and its degradation consequently activates mTOR, independently of the PI3K/AKT/TSC1-2 pathway [55]. This observation provides an additional molecular mechanism to explain the oncogenic activity of 2-HG in AML, as the resulting phosphorylation of p70S6K and 4E-BP/eIF4E36 is responsible for the increased tumor cell size [55], partially counteracted by rapamycin-based treatments [55]. Moreover, mTOR impairs the TCA cycle and then affects the metabolism of cancer cells by controlling mitochondrial activity [56] and stimulating the metabolism of glutamine [57]. The activation of mTOR also contributes to the generation of ROS by altering the electron flow through the electron transport chain (ETC) [58]. Figure 2 depicts the main molecular regulations controlled by 2-HG in AML cells.

The oncogenic effects associated with IDH mutations and high levels of 2-HG were demonstrated in human and murine models. Different studies showed that the expression of human mutants of IDH2 synergistically acts with mutated FLT3 and NRAS to promote leukemogenesis by accumulating undifferentiated cells of the myeloid lineage in the hematopoietic compartment of mice [59,60]. However, although the expression of mutant IDH1/2 both in vitro and in vivo leads to the accumulation of undifferentiated hematopoietic cells [37,39,59,61], it is not sufficient to drive leukemia development and in fact needs the co-presence of high levels of 2-HG, which bias these cells toward the myeloid lineage [62]. These observations are also supported by preliminary results obtained using inhibitors of IDH mutants, which promote the differentiation of leukemic cells by blocking the IDH neomorphic activity and then the production of 2-HG, showing substantial anti-leukemic effects in vivo [61,63,64].

### 2.3. 2-HG as Potential Diagnostic and Prognostic Biomarker

Moreover, identifying high levels of 2-HG in AML patients’ sera suggests that it could also be eligible as a biomarker for diagnosis, prognosis, and as a monitoring tool in AML. Results from different studies reported that the presence of IDH mutations positively correlates with elevated serum 2-HG levels [65,66] and that a concentration of 2-HG higher than 700 ng/mL can predict the IDH mutational status in AML [65], supporting the relevance of the detection of this metabolite to stratify patients who are eligible for specific therapies. Moreover, Wang and colleagues analyzed a cohort of 367 AML patients and observed higher levels of 2-HG in M0/M1 subtypes, suggesting an inverse correlation between the concentration of this metabolite in serum and the degree of differentiation of AML cells [66]. Additionally, changes in 2-HG levels following conventional chemotherapy treatment are reported for IDH-mutated AML [65,67], with a reduction in newly diagnosed patients treated with conventional cytotoxic chemotherapy and subsequent increase upon relapse [68].

High levels of circulating 2-HG are also associated with tumor burden [65] as well as with poor OS and event-free survival (EFS), supporting a prognostic role of this metabolite [66]. Consistently, IDH-mutated patients who show a reduction in 2-HG circulating levels (<200 ng/mL) at CR have improved OS, suggesting that 2-HG testing may represent a sensor for the detection of minimal residual disease (MRD) during clinical remission [65,68].

## 3. 2-HG and the Response of AML to Therapies: Insights from the Clinic

### 3.1. The Role of 2-HG in the Response of AML Cells to Chemotherapy

The generation of 2-HG through the neomorphic activity of mutated IDH enzymes leads to the consumption of NADPH, altering the redox homeostasis of AML cells harboring these mutations. Thus, in principle, AML cells with mutant IDH should be more sensitive to pharmacological strategies aimed at increasing the levels of ROS. It is worth noting that the standard chemotherapeutic regimen used to treat AML induction therapy comprises well-known ROS inducers: cytarabine [69] and an anthracycline [70]. In this scenario, Wang and colleagues demonstrated that mutated IDH1 sensitizes cancer cells to erastin-induced ferroptosis, a non-apoptotic form of cell death caused by excessive accumulation of lipid peroxides [71]. Interestingly, degradation of 2-HG through overexpression of D-2-HG dehydrogenase in the fibrosarcoma cell line HT-1080 inhibited erastin-induced ferroptosis, indicating that this effect is mediated directly by 2-HG rather than by the decrease of NADPH levels. In this context, the authors observed that 2-HG decreased the levels of glutathione peroxidase 4 (GPX4), thus blunting the scavenging of lipid ROS [71]. However, although IDH1/2 mutations are associated with better prognosis of glioblastoma patients [48], their impact on prognosis and response of AML to treatment is still unclear. Several clinical studies reported very poor outcomes of IDH-mutated AML patients [72,73,74], suggesting that AML cells with mutated IDH could be more resistant to intensive chemotherapy. Other studies, however, indicated no differences in term of OS or EFS upon treatment with intensive chemotherapy in patients with IDH mutations compared to patients with wild-type IDHs, or indicated a context-dependent negative prognostic role of increased 2-HG levels [75,76]. In line with the latter observations, Brunner and colleagues studied the role of IDH1 and IDH2 mutations in the response of AML patients to induction therapy and found no differences in CR rates, OS, and EFS between IDH-mutated and wild-type patients, nor a correlation between baseline 2-HG levels and remission rates [77]. Nevertheless, the authors observed the low IDH1/2 variant allele frequency (VAF) correlated with better response to chemotherapy [77], suggesting that high mutation burden could confer resistance to induction therapy in AML cells. Several studies highlight how the prognostic role is influenced by the genetic context and the type of IDH mutations [76,78,79,80]. Patients with concomitant IDH2^R140^ and DNMT3A mutations have a significantly worse prognosis than patients with coexisting NPM1 mutation. In contrast, the association of IDH1^R132^ and DNMT3A mutations does not affect prognosis. In a recent meta-analysis, IDH1 mutations were associated with a lower chance of CR, worse OS, and EFS, especially in patients with normal karyotype. In contrast, IDH2 mutations were associated with favorable outcomes regardless of the type of mutation [81]. Another meta-analysis performed on a very large number of AML patients revealed that IDH1 mutations are associated with worse treatment outcomes in cytogenetically normal AML but not in AML with molecular aberrations [82]. These observations suggest that high levels of 2-HG could predict the response to induction therapy in AML patients, but major genomic aberrations may overcome the dependence of AML cells on 2-HG to induce differentiation blockade and resistance of AML blasts to chemotherapy.

Contrarily, other studies demonstrated an increased sensitivity of cancer cells with IDH mutations to several drugs, including alkylating agents [83], inhibitors of nicotinamide phosphoribosyltransferase (NAMPT) [84], and inhibitors of poly ADP ribose polymerase (PARP) [85].

Chen and colleagues reported that 2-HG released from IDH-mutated AML cells induces the activation of NF-κB pathway in bone marrow stromal cells, thus creating a supportive niche for leukemic cells [86]. The authors observed that 2-HG leads to ROS-induced ERK activation and PIN1-mediated stabilization of p65, which promotes the expression of several NF-κB target genes, among which are cytokines and adhesion molecules. IL-6 released by 2-HG-stimulated stromal cells promoted the proliferation of AML cells, while cell–cell adhesion through VCAM-1 promoted chemoresistance of AML cells [86]. These results suggest that high levels of 2-HG, although not affecting or even enhancing chemosensitivity of circulating AML cells, could promote proliferation and chemoresistance of AML cells in bone marrow (where leukemic stem cells reside), thus fueling the onset of relapse upon therapy discontinuation.

The inconclusive results on prognostic impact of IDH mutations are probably due to differences in population, type of study, and influence of additional genomic aberrations. Large, multi-centric clinical studies are needed to drive a conclusion about the role of 2-HG in the response of AML to chemotherapy. This notion could aid clinicians in stratifying patients that will benefit from induction therapy and patients that, instead, need alternative regimens, such as IDH-targeted therapies.

### 3.2. 2-HG and the Response of AML Cells to Venetoclax

Experiments performed in primary human AML cells demonstrated that mutations in IDH1 or IDH2 genes induce dependence of AML cells on the antiapoptotic protein BCL-2 and, thus, higher sensitivity to venetoclax [87], a specific BCL-2 inhibitor that is already approved by the FDA for the treatment of AML in combination with hypomethylating agents, such as azacitidine [88,89]. Accordingly, clinical trials also confirmed a higher CR rate in AML patients with IDH1 or IDH2 mutations upon treatment with venetoclax and HMA. This sensitization effect is attributable to the inhibitory activity of 2-HG on cytochrome c oxidase (COX), an enzyme that catalyzes the last transfer of electrons in the ETC [90], thus increasing the dependency of cells on BCL-2 to avoid mitochondrial outer membrane permeabilization and apoptosis [87]. Moreover, 2-HG blocks CdC42, a Rho family member that, by interacting with mixed lineage kinase 3 (MLK3), triggers the apoptotic cascade MLK3/MKK4-7/JNK/Bim. In this context, 2-HG blocks apoptosis and promotes cancer cell proliferation by impairing the CdC42-MLK3 axis [91].

The role of 2-HG in AML response to venetoclax was also assessed both ex vivo and in xenograft models. The introduction of octyl-(R)-2-HG, a cell-permeable precursor of 2-HG, significantly reduces the half-maximum inhibitory concentration (IC_50_) of venetoclax in AML cells, with greater effects observed in cells expressing IDH1^R132H^ mutation, compared to those with wild-type IDH1 [87]. Consistently, the same treatment did not induce cell death in healthy cord blood CD34^+^ hematopoietic stem and progenitor cells (HSPCs, used as control), which are also highly resistant to venetoclax treatment ex vivo, suggesting a wide in vivo therapeutic index [87]. The finding that venetoclax could be more effective on IDH mutant cells represents a turning point in the prognosis of AML as the stability of IDH mutations during disease evolution suggests that a population of IDH1/2 mutant cells can survive initial chemotherapy and contribute to relapse [92,93].

## 4. IDH-Targeted Therapies

### 4.1. Ivosidenib and Enasidenib

The recurrence of neomorphic mutations in IDH1 and IDH2 genes in AML and their role in leukemogenesis prompted the development of several specific inhibitors targeting either IDH1 or IDH2 mutant enzymes. Among the compounds that entered in clinical trials, ivosidenib (AG-120) and enasidenib (AG-221) are the only drugs approved by the FDA for the treatment of IDH1- and IDH2-mutated R/R AML, when intensive treatment is precluded [94]. However, these inhibitors have not yet received the approval from the European Medicines Agency (EMA), thus limiting their commercialization in the European Union.

Ivosidenib is the first inhibitor of mutant IDH1 that achieved an anticancer efficacy in clinical trials [95]. It inhibits several mutants at R132 in *IDH1* gene, thus decreasing 2-HG levels and inducing blast differentiation. Ivosidenib efficiently killed IDH1^R132^ mutant cancer cell lines at low nanomolar IC_50_ in vitro, drastically and rapidly reduced 2-HG levels in a mouse xenograft model derived from HT-1080 fibrosarcoma cell line and led to the differentiation of IDH1-mutated AML cells from patients ex vivo [95]. As single agent, in a phase 1 study enrolling 268 R/R IDH1-mutated AML patients ineligible for standard therapy, ivosidenib induced an overall response rate (ORR) of 41.6%, with a duration of CR of 9.3 months [96]. Ivosidenib-based combinations were also tested in clinical trials. DiNardo and colleagues observed that ivosidenib combined with azacitidine in treatment-naïve IDH1-mutated AML patients achieved an ORR of 78% [97]. Moreover, ivosidenib combined with induction therapy led to a CR/CRi/CRp (CR with incomplete neutrophil or platelet recovery) in 88% patients with de novo AML and 50% patients with secondary AML [98]. Recently, early results of phase 3 AGILE trial (NCT03173248) [99] in a front-line setting showed a significantly better outcome in the ivosidenib + azacitidine group than in the azacitidine alone group, with a favorable safety profile. The estimated probability that a patient would remain event-free at 12 months was 37% in the ivosidenib + azacitidine group, compared to 12% in the azacitidine alone group. The median OS was 24 months in the experimental arm compared to 7.9 months of azacitidine group. CR rate was significantly higher in the ivosidenib + azacitidine arm.

Enasidenib reduced 2-HG levels in a IDH2^R140Q^-expressing erythroleukemia cell line, inhibited growth factor-independent proliferation, and reversed hypermethylation of histone H3. Moreover, this compound reduced 2-HG levels, induced differentiation of primary IDH2-mutated AML blasts ex vivo and improved the survival of mice bearing an aggressive xenograft established from an AML patient harboring IDH2^R140Q^ mutation [100]. Stein and colleagues published in 2017 the results of a phase 1/2 dose-escalation and expansion clinical trial evaluating enasidenib as single agent in 239 patients with advanced myeloid malignancies, mostly R/R AML [101]. Among these patients, the ORR was 40.3% and 19.3% patients attained CR, with a median survival of 19.7 months [101]. Interestingly, during this study the persistence of mutated IDH2 allele was observed in peripheral blood mature neutrophils of AML patients despite clinical response, indicating that enasidenib induces the differentiation of blasts rather than their apoptosis. The phase 3 randomized, open-label IDHENTIFY trial (NCT02577406) compared the efficacy of enasidenib monotherapy to conventional therapies, including azacitidine, low-dose, and intermediate-dose cytarabine, in patients with R/R IDH2-mutated AML. Despite the significant gain in CR rate, the survival advantage appeared to be more evident in later follow-ups, and the primary end-point was therefore not fully reached [102]. The combination of enasidenib and azacitidine appears to be effective in a phase 1b/2 study on newly diagnosed IDH2 mutated AML patients with ORR of 74% in the enasidenib + azacitidine group and 36% in the azacitidine monotherapy group. As in the IDHENTIFY study [103], an advantage in terms of survival seems to be more evident in later follow-ups. Recently, Kim and colleagues demonstrated that the combination of enasidenib with all-trans retinoic acid (ATRA)–used to induce differentiation in promyelocytic leukemia (APL) cells [104]–has a synergistic effect on differentiation of a IDH2-mutated cell line and primary IDH2-mutated AML cells ex vivo [105]. Moreover, the combination also induced the differentiation of AML cells from four patients with mutated IDH2 more efficiently than enasidenib or ATRA alone [105]. Based on this preclinical evidence, future clinical trials should evaluate whether the addition of ATRA could prove beneficial in patients with IDH2-mutated AML that do not respond to enasidenib alone. Very recently, a phase 1 open-label study tested ivosidenib or enasidenib in combination with induction chemotherapy in patients with either IDH1- or IDH2-mutated newly diagnosed AML [98]. The rate of CR/CRi/CRp was 77% in the ivosidenib-treated cohort and 74% in the enasidenib-treated cohort, with a 12-month survival probability of 78% for ivosidenib-treated patients and 76% for enasidenib-treated patients [98]. Interestingly, no drug-related deaths were reported and the use of ivosidenib and enasidenib did not seem to affect the time to hematological recovery after induction. The phase 1b/2 clinical trial AG221-AML-005 demonstrated that the combination of enasidenib with azacitidine significantly improved ORR compared to azacitidine alone [103].

Jasra and colleagues recently reported the case of a patient affected by IDH2-mutated AML refractory to induction therapy, who achieved CR upon combined treatment of enasidenib and azacitidine. At relapse, the patient received also venetoclax in combination with the other two drugs, achieving clearance of peripheral blasts [106]. In a phase 2 trial on enasidenib and azacitidine combination in patients with IDH2 mutated AML who are not eligible for intensive treatment, the association of enasidenib, azacytidine, and venetoclax was tested in 11 patients (4 with newly diagnosed AML and 7 with R/R AML) with a CR rate of 86% [107]. These results suggest that the combination of enasidenib, hypomethylating agents, and venetoclax could be an effective treatment option for patients with refractory IDH2-mutated AML. A phase 1b/2 clinical trial is currently evaluating the combination of ivosidenib and venetoclax, with or without azacitidine, in patients with IDH1-mutated AML. The first results, relative to 18 patients, showed a CR rate of 100% with ivosidenib and 800 mg venetoclax [108]. The ongoing phase 1b/2 clinical trial Enaven-AML is evaluating the safety and efficacy of the combination of enasidenib and venetoclax, with promising preliminary results [109].

### 4.2. Other IDH Inhibitors

Besides ivosidenib and enasidenib, several other IDH inhibitors were developed and tested in preclinical and clinical studies. BAY1436032 is an allosteric inhibitor targeting all IDH1 mutations, which inhibits cell proliferation and self-renewal of leukemia stem cells [110]. The combination of BAY1436032 with azacitidine exerts a strong antileukemic activity towards leukemia stem cells both ex vivo and in vivo through the inhibition of the MAPK/ERK and RB/E2F pathway [111]. A phase 1 clinical trial demonstrated a modest effectiveness of this drug, with low ORR and incomplete target inhibition, thus preventing further clinical development [112]. Another IDH1 inhibitor is IDH305, which demonstrated to have promising antitumor activity in IDH1-mutated AML in a phase 1 clinical trial [113]. However, IDH305 is not under clinical development in hematologic malignancies. The most promising second-generation IDH inhibitor is olutasidenib (FT-2102), an inhibitor of mutated IDH1. As a single agent, in a phase 2 clinical trial, olutasidenib induced an ORR of 46% in patients with IDH1-mutated R/R AML, but the presence of co-mutations (*NPM1*, *DNMT3A*, *ASXL1,* and receptor tyrosine kinase (RTK) genes) was correlated with decreased ORR and CR [114]. In combination with azacitidine, olutasidenib induced durable CR in a subset of high-risk AML patients with mutated IDH1 and a 56-day transfusion independence in most patients [115]. Another promising drug is the dual IDH inhibitor vorasidenib (AG-881), which, however, is currently under clinical development only for the treatment of glioma [116].

### 4.3. Differentiation Syndrome and Mechanisms of Resistance to IDH Inhibitors

When using IDH inhibitors in the clinic, two main drawbacks should be taken into account: (i) severe adverse effects exist; (ii) several mechanisms are responsible for the resistance of AML cells to these drugs. A serious adverse effect induced by IDH inhibitors is differentiation syndrome (DS), a potentially lethal complication also induced by ATRA in APL [117]. DS is characterized by a plethora of symptoms associated to inflammation (driven by differentiation and expansion of granulocytes), such as dyspnea, fever, hypotension, and pulmonary infiltrates [117]. In 2016, Birendra and DiNardo reported the cases of three patients with R/R IDH1-mutated AML treated with ivosidenib, showing fever, chills, dyspnea, and pleuro-pericardial effusions [118]. The clinical trials that led to the approval of IDH inhibitors by the FDA revealed that 11–14% patients with R/R AML develop DS [97,101], which is managed with corticosteroids, hydroxyurea, and temporary discontinuation of IDH inhibitors. The relation between the onset of DS and clinical outcome is controversial. Indeed, Fathi and colleagues reported an ORR of 45.5% in enasidenib-treated AML patients who experienced DS, while the ORR in patients without DS was 37.5% [119]. However, Norsworthy and colleagues, by analyzing a cohort of 179 AML patients receiving ivosidenib and a cohort of 214 AML patients receiving enasidenib, reported an inferior outcome for patients who developed DS [120]. Further clinical data are required to drive conclusions regarding the role of DS on the clinical outcome of IDH inhibitors-treated AML patients.

Both primary and secondary mechanisms of resistance to IDH inhibitors have been described. Co-occurring mutations in RTK pathway genes, such as *NRAS*, *KRAS*, *PTPN11*, and *FLT3*, are associated with primary resistance to ivosidenib despite the reduction in 2-HG levels [121]. Moreover, the onset of KRAS mutations was observed in patients treated with ivosidenib at relapse or progression [121]. Secondary resistance to ivosidenib is also characterized by the onset of 2-HG restoring mutations, such as second-site mutations in the *IDH1* gene and canonical mutations of *IDH2*, which can co-occur in ivosidenib-resistant AML [121]. Quek and colleagues used single-cell whole genome sequencing to study the mechanisms of relapse in 37 IDH2-mutated R/R AML patients treated with enasidenib. The authors found no second-site *IDH2* mutations but reported seven different patterns of clonal evolution, including mutations of *IDH1*, cytokine receptor signaling genes, hematopoietic transcription factors, components of the spliceosome, and chromosome 7 monosomy, which restored the differentiation blockade in AML cells [122]. As isoform switching between IDH1 and IDH2 mutations is a mechanism of acquired resistance to specific IDH inhibitors through the restoration of 2-HG levels [123], combined inhibition of both IDH isoforms or the use of dual IDH1/2 inhibitors, such as vorasidenib, could be a viable strategy to avoid the increase in 2-HG levels in AML cells. Beyond these genetic mechanisms, Stuani and colleagues recently observed that IDH mutant cell lines, patient-derived xenografts, and AML cells from patients exhibit a sustained mitochondrial metabolism (OXPHOS), with increased respiration and methylation-driven CEBPα-induced fatty acid β-oxidation (FAO) [124]. Interestingly, ivosidenib reduced 2-HG levels without affecting FAO and OXPHOS. However, OXPHOS inhibitors increased the anti-leukemic efficacy of IDH inhibitors in vivo, indicating that mitochondria-targeted therapies could overcome the resistance to IDH inhibitors [124].

## 5. Concluding Remarks

From an epidemiological point of view, AML represents a global health issue. In fact, among leukemias, it has the highest lethality rate worldwide [8]. Moreover, most AML patients relapse or are refractory to the standard therapies. Elderly or unfit patients cannot withstand intensive induction therapy and are thus prone to relapse. Indeed, consolidation therapy and allo-HSCT are fundamental steps to obtain a complete remission of the disease. These notions underscore the importance to find new diagnostic and therapeutic options for these otherwise incurable patients.

The identification of specific genetic alterations could broaden the therapeutic scenario, as most mutations are associated to different prognostic outcomes. Indeed, the FLT3-ITD mutations predict a worse prognosis for patients through the activation of several oncogenic pathways (e.g., RAS/MAPK, STAT5, and PI3K/AKT) [11]. On the other hand, mutations that affect NPM1 are favorable in terms of OS and disease-free survival (DFS) and are associated with lower incidence of relapse [2]. However, when co-occurring, these two genetic alterations determine a high risk of a worse clinical course for patients, underlying the crucial role of these genes in AML development and progression.

The most interesting mutations reported to have a great impact on AML affect the NADP-dependent isocitrate dehydrogenases IDH1 and IDH2. These mutated enzymes acquire the ability to convert α-KG to 2-HG, a oncometabolite that accumulates in AML cells [23], induces epigenetic deregulations by inhibiting α-KG-dependent enzymes [24], and alters cell metabolism, leading to the activation of oncogenes and the inactivation of tumor-suppressor genes. The accumulation of 2-HG is a common event of many types of cancers, and high levels of this oncometabolite are associated to worst prognosis [26]. Indeed, 2-HG promotes an increase in ROS levels by slowing down mitochondrial respiration and altering the electron flow across the ETC [47], thus increasing the aggressiveness of cancer cells [58]. However, elevated ROS levels make these cells more sensitive to oxidative stress. Increased ROS levels are also a consequence of the activation of the mTOR pathway [58] by 2-HG, which promotes the accumulation of undifferentiated myeloid cells [59,60] and boosts anabolic processes [56,57], thus favoring leukemogenesis. Moreover, the oncogenic activity of 2-HG is sustained by the repression of the HIF pathway, which leads to the suppression of the immune response against tumor cells in T-cells [54].

Although all these observations support the role of 2-HG as a tumor-promoting molecule, its role in the response of AML blasts to therapies is less clear. Unlike other IDH-mutated tumors such as gliomas, AML cells with high levels of 2-HG were reported to be either more resistant to induction therapy or more sensitive to several other therapies like PARP inhibitors [85] or venetoclax [87]. Regarding induction therapy, several clinical studies indicate that several parameters, such as the presence of complex genomic rearrangements, could influence the role of 2-HG in the response of AML cells [82]. However, it is worth noting that the paracrine release of 2-HG by leukemic cells–besides suppressing the immune response through the degradation of HIF in T-cells–could affect stromal cells in the bone marrow, leading to the formation of a supportive niche that promotes chemoresistance of AML [86]. This observation sustains a key role for 2-HG in the onset of chemoresistance in IDH-mutated AML cells and highlights the importance of the crosstalk between leukemic and healthy cells in tumor microenvironment. Future clinical studies should determine whether elevated 2-HG levels could predict the response to targeted therapies of AML cells with co-occurring mutations.

Given its pathogenic role in AML, 2-HG could be exploited as a molecular target to counteract tumor relapse in IDH mutant cells. Indeed, circulating 2-HG was found to be associated with tumor burden [65], poor OS, and EFS, defining it as a negative prognostic factor [66]. In this scenario, the development of pharmacological inhibitors of mutated IDH enzymes changed the outlook for AML patients with these genetic alterations. Interestingly, through the sustained reduction in 2-HG levels, IDH inhibitors lead to the differentiation of IDH1- or IDH2-mutated blasts, respectively. It is reasonable that the combined action of blast differentiation by IDH inhibitors–eventually enhanced by other differentiating agents [105]—and cell death induction by chemotherapy could induce a potent antileukemic effect. Indeed, the combination between standard induction therapy or hypomethylating agents and IDH inhibitors, such as ivosidenib and enasidenib, demonstrated to improve the OS and CR of AML patients by leading to the differentiation of AML cells, which became more sensitive to treatments. In line with these observations, recent clinical data indicate that novel combined therapies based on the inhibition of mutated IDH enzymes and induction of apoptosis in cancer cells–through, for instance, the addition of venetoclax [106,108]—could achieve very high CR rate in R/R AML patients. The deep knowledge of the molecular alterations accounting for chemoresistance in AML cells could aid clinicians in the design of novel, rational combination therapies to bypass these mechanisms, thus leading to blast eradication. In this context, targeting the molecular determinants of resistance to IDH inhibitors, such as mutated FLT3, with targeted molecules promises to be an effective strategy to overcome primary resistance in patients and achieve high response rates. A phase 1b clinical trial (NCT04655391) will start recruiting patients in June 2022 to evaluate the combination of ivosidenib and enasidenib with glasdegib, an inhibitor of Sonic Hedgehog, which is involved in the persistence of leukemic stem cells [125]. Moreover, the discovery that increased mitochondrial metabolism contributes to the resistance of AML cells to IDH inhibitors [124] could in the future pave the way for the development of innovative therapies based on the combination of IDH inhibitors with drugs that impair mitochondrial respiration, such as the antidiabetic metformin [126].

In conclusion, 2-HG has a great potential both as a prognostic marker in IDH-mutated AML and as a biomarker of clinical outcome to therapies in AML patients. Moreover, the potent antileukemic effect achieved through the pharmacological reduction in its levels indicates that 2-HG is a therapeutic target in IDH-mutated AML. Further studies should be carried out to dissect in detail the complex effects of 2-HG on leukemic cells and on healthy bone marrow cells, and its role in the response of AML blasts to therapies, thus improving the management of R/R AML with mutated IDH enzymes.
